# Construction and validation of the Self-care Assessment Instrument for patients with type 2 diabetes mellitus**^1^**

**DOI:** 10.1590/1518-8345.1533.2890

**Published:** 2017-06-05

**Authors:** Simonize Cunha Barreto de Mendonça, Maria Lúcia Zanetti, Namie Okino Sawada, Ikaro Daniel de Carvalho Barreto, Joseilze Santos de Andrade, Liudmila Miyar Otero

**Affiliations:** 2 MSc, RN, Hospital Universitário de Sergipe, Aracaju, SE, Brazil.; 3 PhD, Associate Professor, Escola de Enfermagem de Ribeirão Preto, Universidade de São Paulo, WHO Collaborating Centre for Nursing Research Development, Ribeirão Preto, Brazil.; 4 Doctoral student, Universidade Federal Rural de Pernambuco, Recife, PE, Brazil.; 5 PhD, Adjunct Professor, Departamento de Enfermagem, Universidade Federal de Sergipe, Aracaju, SE, Brazil.; 6 PhD, Associate Professor, Departamento de Enfermagem, Universidade Federal de Sergipe, Aracaju, SE, Brazil.

**Keywords:** Self Care, Diabetes Mellitus, Type 2, Psychometrics, Validity of Test

## Abstract

**Objective:**

to construct and validate the contents of the Self-care Assessment instrument for patients with type 2 diabetes mellitus.

**Method:**

methodological study, based on Orem's General Theory of Nursing. The empirical categories and the items of the instrument were elucidated through a focus group. The content validation process was performed by seven specialists and the semantic analysis by 14 patients. The Content Validity Indices of the items, ≥0.78, and of the scale, ≥0.90, were considered excellent.

**Results:**

the instrument contains 131 items in six dimensions corresponding to the health deviation self-care requisites. Regarding the maintenance, a Content Validity Index of 0.98 was obtained for the full set of items, and, regarding the relevance, Content Validity Indices ≥0.80 were obtained for the majority of the assessed psychometric criteria.

**Conclusion:**

the instrument showed evidence of content validity.

## Introduction

Diabetes mellitus (DM) stands out among the chronic diseases due to its high prevalence and its impact on morbidity and mortality indicators in the national^1^ ^-^ ^2^ and global contexts^3^. The concept of self-care in DM is related to a variety of factors, ranging from maintaining a healthy diet, self-monitoring of blood glucose, use of medications, regular physical activity, foot care, healthy coping and risk reduction^4^ ^-^ ^5^. From this perspective, the implementation of strategies aimed at the self-management of the disease and the encouragement of self-care is critical.

Structured education for the self-management of type 2 diabetes mellitus (DM2) is a strategic resource to equip patients for making decisions in relation to the treatment. A review study of the educational process showed positive results for the self-management of DM2. These results relate to the support provided for the self-management of the disease and the continuous monitoring in the control of blood glucose, as well as for the prevention of acute and chronic complications^6^.

It is recognized that the multidisciplinary health team should promote the development of self-care skills in order to make people with DM co-responsible for the requirements of their daily life, with regard to the treatment, modifying or maintaining healthy habits and strengthening self-confidence^7^ ^-^ ^8^. Thus, self-care should be understood as learned behavior that is performed by individuals for their own benefit^9^_._

In this sense, the evaluation of self-care actions taken by patients with DM2 should be integrated into the care provided by the health professionals. The use of instruments that measure self-care actions constitutes a methodological tool that assists in the evaluation of the responses of the patients to treatment, allowing the comparison of data over time and the understanding and study of the problems observed^10^, in addition to guiding behavior in the clinical practice.

There are instruments for self-care assessment described in the literature^10^ ^-^ ^13^, however, these do not cover the multidimensionality of the disease, and are mostly directed toward the evaluation of adherence to the medication therapy, not including seeking multidisciplinary care, knowledge about the disease and discomforts of the treatment or the process of acceptance of the disease. Systematic review studies^14^ ^-^ ^15^ ^)^ have highlighted the lack of instruments for the assessment of self-care behavior in people with DM2.

Given this gap and considering the lack of instruments based on the theoretical model of self-care suggested by Dorothea Orem^9^, the development of an instrument based on the health deviation self-care requisites was proposed. This theoretical model has been used as the theoretical and philosophical basis to support the practice of Nursing in a variety of situations, with an emphasis on the care of patients with chronic diseases^16^. The Orem assumptions fit the purpose of this study, as they cover promotion and education actions, with individuals encouraged to take responsibility for the care of their own health.

The construction of a measurement tool based on the theoretical model of Orem^9^ has been shown to be relevant to enable health professionals to develop integral care strategies for patients with DM2, through observation and transformation of the clinical practice, especially affecting the planning of nursing care. Given the above, this study aimed to construct and validate the contents of the Self-care Assessment Instrument for patients with type 2 diabetes mellitus (INAAP-DM2).

## Method

Methodological study, which adopted the psychometric procedures^17^ for the preparation of measurement instruments as a reference, which include three specific poles (theoretical, empirical and analytical). In this study, the theoretical pole was developed, which covered the construction and content validation of the INAAP-DM2.

Initially, the aim was to deepen the knowledge about the self-care construct of DM2 patients, culminating in the choice of Orem's General Theory of Nursing^9^ to support the design of the domains and items that would compose the instrument. The comprehension of this theoretical model is linked to the concept of self-care as the practice of activities undertaken by individuals for their own benefit. In the presence of any health problems, the implementation of these activities will be linked to specific requisites with the intention of recovery, rehabilitation and control. The six self-care requisites in conditions of disease defined by Orem (Seeking and securing appropriate multidisciplinary care; Being aware of and attending to the disease and its complications; Adhering to the treatment; Being aware of and considering/regulating the discomforts of the treatment; Accepting the disease and the need for health care and learning to live with the effects of the disease and the consequences for the lifestyle of the medical diagnosis and treatment measures) were assumed to be the theoretical dimensions of the construct, with the empirical categories elucidated through the focus group technique^18^ ^-^ ^19^, in the months of May and April 2015.

**Figure 1:**
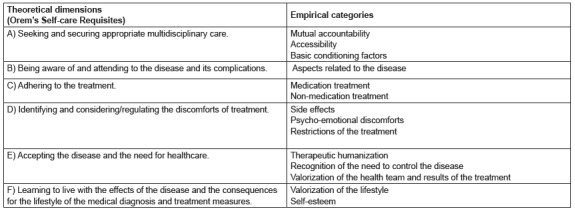
Theoretical dimensions and empirical categories of the instrument. Aracaju, SE, Brazil, 2015

Three separate focal groups were formed, one by professionals experienced in management of patients with DM2 and the other two composed of DM patients enrolled in an educational program of an outpatient service of reference in the state of Sergipe. The discussions of the participants followed a script composed of questions based on the six health deviation self-care requisites. The textual corpus was formed from the discussions that emerged in the sessions, which were audio recorded and transcribed in full, with subsequent division of the text into the six theoretical dimensions with their respective empirical categories (Figure 1). 

Each theoretical dimension represented a domain and was validated through the actions that reflect the self-care identified in the focal group technique. It should be noted that the items of dimension C that refer to adherence to the treatment with oral hypoglycaemic agents and insulin, were adapted from the Measure of Adherence to Treatment (MAT)^20^ instrument, as the self-care actions mentioned in the focus groups were matched and submitted to the validation process. A Likert-type scale with five points was chosen to represent the numerical items, with number "1" being equivalent to the worst score and number "5" the best.

The instrument contains items with a scale of frequency - *never, almost never, sometimes, often* and *always* and a scale of knowledge - *do not know, answers 1 item, answers 2 items, answer 3 items* and *answers more than 3 items.* After the application of the instrument, at the end of each domain, the score should be added and divided by the number of items applied, resulting in a partial score. The partial score of each self-care requisite will result in the classification of the Nursing Systems^9^: *Wholly Compensatory* (score 1 or 2) - the patient is unable to engage in therapeutic self-care actions; *Partly Compensatory* (score 3) - the patient is able to learn, but needs professional and/or family to perform the self-care actions and *Supportive-Educative* (score 4 or 5) - the patient is able to learn and perform the therapeutic self-care actions alone.

After the design of the items, the first version of the instrument and the instruction manual were sent, via email, to seven diabetes specialists, requesting the content validation^17^. The specialists were selected from the database of the Coordination for the Improvement of Higher Education Personnel - CAPES, with the inclusion of those who obtained a minimum score of five points, according to the adapted criteria for the selection of specialists^21^. There was no restriction regarding the participation of different professional categories, with the selection of those whose academic profile revealed expertise in the construct that the instrument was intended to measure.

The questionnaire for the analysis of the instrument was made available in two formats: MSWord and Google docs electronic form. Thus, the assessment of the items was carried out considering the domain to which they belonged, their maintenance in the instrument and the presence of the psychometric criteria of: objectivity (to express desirability or preference), simplicity (to express a single idea), clarity (to be intelligible even to the lowest strata of the population), relevance (to be consistent with the attribute to be measured), accuracy (to be distinguished from the other items), modality (not to use extreme expressions), typicality (to use typical expressions for the attribute) and credibility (not to sound ridiculous, unreasonable or infantile)^17^. In addition, there was a space for suggestions from the experts.

The level of concordance among the specialists was previously defined, considering excellent as Content Validity Index of the Items (CVIi) greater than or equal to 0.78 and the mean CVI of the scale (CVIs) of 0.90 or greater^22^. To calculate the CVIi, scores of "1" to "3" were assigned, respectively, to the responses *maintain without changes, maintain with changes* and *do not maintain*. The numerator corresponded to the sum of the "1" and "2" responses, and the denominator to the total number of experts. To evaluate the number of items in each domain and the total number of items of the instrument, the mean of the CVIi was used, calculated separately and divided by the number of items considered in the evaluation. For the analysis of the items, regarding the relevance to the domains and the psychometric criteria, the arithmetic mean was calculated through the sum of the responses "*maintain in the domain*" or "*yes*", respectively, divided by the total number of specialists.

After the adjustments suggested by the specialists the instrument was subjected to semantic analysis, in October 2015, by 14 patients with DM2 enrolled in an outpatient service of reference in the state of Sergipe. The application of the instrument was performed individually, in a reserved consulting room, with the lowest and the highest stratum of the target population^17^, which took an average of 60 minutes.

The research project was approved by the Human Research Ethics Committee of the Federal University of Sergipe (UFS), under registry number 40789414.8.0000.5546.

## Results

The six dimensions of the self-care construct for DM2 patients included 131 items, 26 relative to dimension A, eight items to dimension B, 63 items to dimension C, 16 items to dimension D, ten items to dimension E and eight items to dimension F. Of the dimensions, only C was divided into sub-dimensions (medication treatment - pills and insulin; non-medication treatment - dietary plan, physical activity plan, blood glucose monitoring and foot care).

These items were submitted to content validation by a committee composed of seven specialists, consisting of one physical educator, three nurses, one doctor, one nutritionist and one psychologist. In this committee there was a predominance of females (71.4%), aged over 50 years (85.7%), with more than 30 years since graduation (85.7%), and the majority had 10 to 15 years of professional experience in DM (57.1%). All the judges had a PhD and expertise to evaluate the construct, as evidenced by their performance of research on issues related to the construct (100%), publication of articles in indexed journals (85.7%), performance of training/specialization courses (85.7%) and recent clinical practice in the DM area (85.7%).

Regarding the judgment of the specialists in relation to the domain in which each item belonged, 129 items had CVIi ≥0.78 and all the domains exhibited CVIs ≥0.90. Item 19 (domain A) and 113 (domain D) presented CVIi of 0.57 and 0.71, respectively, however, both remained in the original domain since they were consistent with their respective self-care requisites. Regarding the maintenance in the instrument, all items presented CVIi ≥0.78, and in domains B and D, all of the items exhibited CVIi of 1.00. The set of items in each domain presented CVIs ≥0.90, namely, domain A (0.99), B (1.00), C (0.98), D (1.00), (0.97) and F (0.96). The full set of items presented CVIs of 0.98 evidencing satisfactory content validity.

The evaluation of the specialists resulted in the indication of the maintenance of all items, however, 65 of them (49.6%) presented CVIi less than 0.78 relative to maintaining them unchanged, indicating the need for redesign. When considering the distribution by domain of the items that needed to be redesigned, this showed: A (24 items), B (4items), C (32 items) and F (5 items). Grammatical modifications were carried out, as well as the substitution of negative terms and words that were difficult for the lower strata of the population to understand.

Regarding the relevance of the items to the psychometric criteria^17^, the evaluation of the specialists was satisfactory, since the domains presented CVIs ≥0.80 for the majority of the criteria evaluated (Table 1).

**Table 1:** Content Validity Indices of the scale obtained from the evaluation of the judges regarding to the relevance of the domains to the psychometric criteria. Aracaju, SE, Brazil, 2015

Psychometric Criteria	A	B	C	D	E	F
Objectivity	0.88	0.89	0.95	0.98	0.96	0.98
Clarity	0.87	0.91	0.89	0.97	0.99	0.84
Accuracy	0.86	0.82	0.89	0.96	0.96	0.91
Typicality	0.82	0.84	0.82	0.85	0.80	0.82
Simplicity	0.86	0.80	0.80	0.85	0.84	0.82
Relevance	0.87	0.93	0.84	0.85	0.84	0.80
Modality	0.80	0.82	0.81	0.85	0.83	0.80
Credibility	0.83	0.84	0.83	0.86	0.81	0.73

Considering CVIi ≥0.78 as excellent, 65 items (49.62%) presented at least one psychometric criterion with lower CVIi. However, in 55 of these, the lowest CVIi was 0.71, which corresponds to approval by five of the seven specialists. Thus, only ten items displayed psychometric criteria with CVIi ≤0.59, with one item in domain A (18), seven items in B (39, 60, 63, 68, 69, 70, 83) and two items in F (124 and 131). Some items presented at least one psychometric criteria with CVIi of 0.71, however, were not redesigned as they exhibited CVIi of 0.86 or 1.00 for the judgment of *maintain without change*, with consequent absence of suggestions for modifications by the panel of specialists. The majority of the suggestions of the specialists were accepted, aiming for better comprehension.

Next, the semantic analysis was conducted with a sample of the target population, with a predominance of females (85.7%), residents of the state capital (85.7%), who were literate (78.6%), although the majority had only five years of education (57.1%). Half of the patients had been diagnosed with type 2 diabetes for over 15 years. The participants reported that they did not have great difficulties of comprehension. The patients with up to five years of study showed difficulty in a mean of 11 items, those with up to 10 years, five items and those with over 10 years, a mean of four items, which confirms the principle that if the lower strata of the population understand the items, so will the rest of the population^17^.

**Figure 2:**
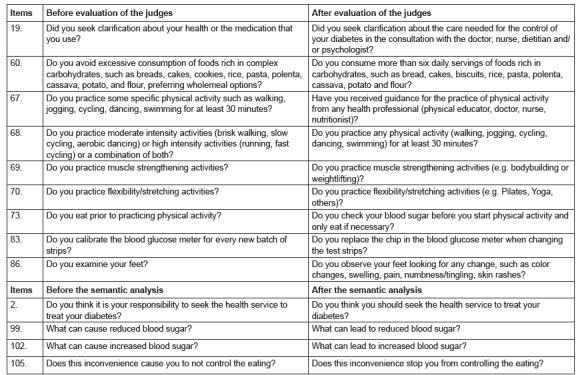
Main changes made to the items of the Self-care Assessment Instrument for Patients with DM2 after the evaluation of the judges and the semantic analysis. Aracaju, SE, Brazil, 2015

From a total of 131 items, only 8, one in domain A (2), four in C (51, 60, 77, 79) and three in D (99, 102, 105) were highlighted as unclear, being redesigned so that the patients could better understand them. Among the changes performed, after the evaluation of the judges and semantic analysis, those conducted on some items stood out (Figure 2).

## Discussion

The construction of a measuring instrument requires the design of the items that behaviorally represent the construct of interest^17^. The choice of Orem's Self-care conceptual model for the theoretical basis of the instrument and the use of the focus group technique allowed the relevant topics to be identified to cover the domains that make up the construct and to promote ideas of how the items should be displayed. Thus, the realization of these groups with health professionals and patients enabled the factors, barriers and difficulties involved in the therapeutic requirement to be contemplated and the construct and the theoretical framework adopted to be better represented.

These aspects were analyzed from the perspective of the six health deviation self-care requisites postulated by Orem^9^. The items of domain A cover the importance of the mutual accountability of the professionals and patients in order to ensure accessibility to health services, as well as including conditioning factors for seeking appropriate care, such as financial status, family support and socio-cultural orientation. In domains B and D the items assess, respectively, the knowledge of the patient related to aspects of the disease (causes, complications, examinations, treatments) and the discomforts of the treatment (medication side effects, psycho-emotional discomforts, eating restrictions). Knowledge and understanding of these aspects should be assessed as they contribute to the self-management of DM^4^ ^-^ ^6^ and consequently relate to better glycemic control^23^. 

Domain C included the self-care practices related to medication^20^ and non-medication treatment (food plan, physical activity plan, blood glucose monitoring and foot care). In domains E and F, the items refer to coping with the disease, addressing the acceptance and the condition of learning to live with the consequences of the treatment. The factors that hinder coping with the disease affect the performance of self-care and should, therefore, be identified by the health team^24^.

The contents and format of the items were reformulated according to the contributions of specialists. The composition of the panel with different professional categories and experiences related to the subject allowed a broad and deep evaluation, with relevant and complementary observations. The results indicated satisfactory content validity, with the full set of items presenting CVIs of 0.98 for being maintained in the instrument. Regarding the psychometric criteria, the domains showed CVIs ≥0.80 for the majority of the evaluated criteria. Some items presented psychometric criteria of 0.71, despite the judgment to maintain them unchanged. This discrepancy may have resulted from difficulties presented by the specialists regarding the evaluation of the psychometric criteria.

The results demonstrated the validity of the content of instrument, however, it must be subjected to the experimental and analytical procedures postulated by the psychometric model, so that it can be used in the clinical practice and scientific studies. From this perspective, the development of a technology based on a theoretical model of nursing demonstrates how this science has to contribute to public health. Furthermore, it is a tool that addresses the dimensions: seeking appropriate multidisciplinary care, adherence to medication and non-medication therapy, knowledge about the disease and the discomforts of the treatment, and acceptance of the disease, considering the importance of the multidimensionality of the integrality of the care.

Understanding these dimensions will facilitate the management of patients with DM2, as the measure will allow the detection of the completion of the self-care requisites. In this study some difficulties were encountered, among which, the number of specialists who agreed to participate and the time taken returning the evaluations stood out.

## Conclusion

This study allowed a better comprehension of the meanings of the self-care requisites, from the perspective of health professionals and DM2 patients, and allowed an instrument to measure this construct to be developed, with evidence of content validity. Future studies are recommended to test its psychometric properties and make it a valid and reliable tool in the assessment of the self-care of DM2 patients, by identifying the requirements for their compliance. This will contribute to decision making in the clinical practice, as well as to obtaining better results in the self-management of the care by the patients.
